# The Selectivity and Suitability of Online Learning Resources as Predictor of the Effects of Self-Efficacy on Teacher Satisfaction During the COVID-19 Lockdown

**DOI:** 10.3389/fpsyg.2022.765832

**Published:** 2022-05-02

**Authors:** Yonghai Zhu, Yingying Xu, Xinyu Wang, Shiyu Yan, Li Zhao

**Affiliations:** ^1^Elementary Education College, Capital Normal University, Beijing, China; ^2^School of Education Science, Nanjing Normal University, Nanjing, China

**Keywords:** online learning resources, teachers’ satisfaction, selectivity of online learning resource, suitability of online learning resource, online teaching self-efficacy, technology self-efficacy

## Abstract

Online learning resources (OLR) play an important role in teaching and learning in the process of online learning. Teachers will be satisfied with selectable and suitable online learning resources, which can promote their self-efficacy to facilitate online teaching and learning. This study proposed a model to examine the effects of the selectivity of online learning resources (SE-OLR) and the suitability of online learning resources (SU-OLR) on teachers’ online teaching satisfaction, and the mediating role of technology self-efficacy (TECHN-SE) and online teaching self-efficacy (OT-SE) between them. The results indicated that SE-OLR and SU-OLR positively affected teachers’ online teaching satisfaction; TECHN-SE and OT-SE positively influenced teachers’ online teaching satisfaction, while TECHN-SE and OT-SE played mediating roles between SE-OLR and SU-OLR and teachers’ online teaching satisfaction. The findings have implications for the design and development of online learning resources to improve teachers’ satisfaction and facilitate students’ learning effectiveness and teachers’ online teaching.

## Introduction

During the COVID-19 lockdown, traditional face-to-face teaching was replaced by online teaching. As an important complement for teachers to implement teaching, learning resources will have a great impact on the whole teaching process and teachers’ instructional performance. For online learning, more learning resources according to the learning content of the textbooks should be provided for learners as there are not enough learning resources for online learning compared with the traditional face-to-face learning. Thus, teachers have to develop online learning resources (OLR) for learners by themselves. Previous studies concluded that teachers’ satisfaction was related to their online teaching performance (Judge et al., 2001; Luca et al., 2019). However, there are a great number of online learning resources for teachers to select and utilize in their design of suitable online learning resources for online learners. Undoubtedly, teachers will be satisfied with suitable online learning resources, and their satisfaction with online learning resources influences their teaching effectiveness and finally improves students’ learning effectiveness. Previous studies have shown that increasing learning satisfaction can encourage learning participation in the online learning environment (Chan et al., 2021). In the same way, improving online teaching satisfaction may also encourage participation in online teaching environments. Therefore, this study took online learning resources as a factor affecting teachers’ satisfaction, and the study on online learning resources focused on two main aspects: the selectivity of online learning resources (SE-OLR) and the suitability of online learning resources (SU-OLR).

Prior to the COVID-19 shutdowns, many teachers had no previous experience of online teaching or learning due to the main form of education still being face-to face learning. Therefore, this large-scale online learning phenomenon was undoubtedly a new challenge for the vast majority of teachers (Hong et al., 2022). Most teachers are unfamiliar with online teaching, which can lead to learning ineffectiveness. However, different teachers show great differences when encountering the same problems in online teaching. For example, a teacher with a higher sense of self-efficacy will exert more effort when facing problems, which then leads to better performance (Malinen et al., 2013; Schipper et al., 2018). On the contrary, a teacher with low self-efficacy is likely to be unwilling to make more efforts when facing problems, and the probability of failure increases accordingly, which leads to the decline of teachers’ satisfaction with online education. Therefore, teachers’ self-efficacy has a significant positive effect on teachers’ satisfaction (Bogler, 2001; Luca et al., 2019). Combined with the conditions of online teaching, in this study, teachers’ self-efficacy was divided into two parts: online teaching self-efficacy (OT-SE) and technology self-efficacy (TECHN-SE), where OT-SE refers to teachers’ self-efficacy about their own professionalism, while TECHN-SE refers to their self-efficacy about technology supporting their online teaching.

In the study of online learning resources and teachers’ self-efficacy, Timothy (2009) came to the conclusion that the improvement of teachers’ online self-efficacy was conducive to the fuller utilization of teaching resources. However, there has been no relevant study of the influence of online learning resources on teachers’ self-efficacy. Thus, a model was constructed in this study to predict the effect of SE-OLR and SU-OLR on teachers’ satisfaction, and the mediation of TECHN-SE and OT-SE, providing a new idea for improving teachers’ satisfaction and thus improving teaching effectiveness, and having some implications for those who design and develop online learning resources.

## Literature Review

### Selectivity and Suitability of Online Learning Resources

Online learning, a broad term to describe learning facilitated by the Internet, has been adopted at educational institutions around the world (Lim et al., 2021). Online learning resources are supported by various technical resources, including computers, mobile devices (e.g., smartphones and tablets), digital cameras, social media and Internet platforms, software applications, etc. With the development of information and communication technology (ICT), online learning has become an inevitable trend. In order to ensure the quality of online education, the reasonable development and utilization of online learning resources is very important. The teacher plays a critical role and is the leader of the whole teaching process. As a result, teachers should make full use of online learning resources to enrich their curriculum content.

There are a myriad of useful online resources to be shared through various channels, and the web is a sea of information that encompasses every imaginable type of content (Kio and Lau, 2017). Teachers have the opportunity to choose the resources they want to assist them in their teaching. For example, they could choose resources provided by some of the most reputable institutions in the world for quality teaching and learning, which can have a positive effect on their satisfaction (Mulhem and Wang, 2020). In online teaching, more online learning resources should be provided for students than in face-to-face learning. Teachers should select, re-organize, and design the learning resources for students according to the learning content of the textbooks. Thus, in this study, SE-OLR refers to the teachers’ ability to specifically select and control online learning resources based on the learning content of the textbooks.

A number of studies have shown that the use of audio-visual learning resources can make learning more effective, especially the use of instructional videos and online lectures (Mitra et al., 2010; Borup et al., 2011; Crook and Schofield, 2017; Scagnoli et al., 2019). The use of audio and visual online learning resources facilitates learning, so teachers need to choose the most suitable form of resources to match the learning content to promote learning effectiveness. In this study, SU-OLR refers to the degree of fit between the existing online learning resources and the teaching materials adopted by teachers for their online instruction. Only the matching of resources and teaching content can make teachers feel the usefulness of resources, thus affecting their online teaching satisfaction and learning effectiveness.

### Technology Self-Efficacy and Online Teaching Self-Efficacy

In social cognitive theory, Bandura proposed the concept of self-efficacy which refers to one’s belief in being able to organize and execute certain actions (Bandura, 1977). Self-efficacy is the ability to influence a person’s preference for a particular domain and his or her behavior. In other words, self-efficacy affects the choice to participate in a task, the effort exerted to perform that task, and the perseverance to complete the task (Bouffard-Bouchard, 1990; Bandura, 1997).

With the development of social psychology, the concept of self-efficacy has been adapted in many fields and applied in different disciplines. When relating self-efficacy to online learning, researchers have approached it from different perspectives (Zhao et al., 2021). Online learning resources are based on the internet, which requires students and teachers to master certain types of information and communication technology (ICT) to make full use of online learning resources. Menon et al. (2017) conceptualized the self-efficacy of technology integration as teachers’ beliefs in their ability to successfully integrate technology in a way that promotes students’ learning. In this study, TECHN-SE refers to teachers’ belief in their ability to access and assess the possibility of using new technology to conduct teaching activities with sufficient support provided by the school to meet their technological needs. It is very important for teachers to make full use of computers and the internet in their teaching because they can access more information and resources through this technology. Thus, teachers are expected to learn to use new applications and technologies to improve their teaching process (Ekizoglu and Ozcinar, 2010). Regarding technology-based behavior, a number of meta-analyses have found that there is a good correlation between perceived behavioral control and the usefulness of certain technologies (Pan, 2020), which means that self-efficacy of technology is related to its usefulness. Therefore, teachers with high TECHN-SE can make full use of the technology to improve their teaching process. Moreover, Timothy (2009) found that teachers’ self-efficacy affects how they use technology in the classroom, meaning that the higher their TECHN-SE, the more motivation they will have when using online resources.

According to Bandura’s definition, self-efficacy is one’s confidence in one’s ability to organize and implement the actions necessary to achieve one’s goals. Self-efficacy belief is the result of individual emotion and the psychological thinking cognitive process (Bandura, 1997). Meanwhile, teaching self-efficacy refers to the belief of teaching faculty in their ability to meet the challenges of teaching and to provide effective teaching for their students (Culp-Roche et al., 2021). In this study, online teaching self-efficacy (OT-SE) could be regarded as the evaluation and judgment of whether teachers are competent to perform online teaching tasks when they carry out online education. The more the knowledge of a subject increases, the more OT-SE of the subject the teacher will have, as well as the more time they will spend on that subject (Brannick et al., 2005). For teachers, high OT-SE means they believe they have sufficient subject knowledge to carry out online teaching. If they have high OT-SE, they will have the confidence to achieve the online teaching goals and will try their best.

The results of Li’s (2019) study showed that there was a positive correlation between teachers’ OT-SE and teachers’ competence; that is, the higher the teachers’ OT-SE, the stronger their competence in online teaching will be. Moè et al. (2010) showed that teachers’ self-efficacy could help improve their job satisfaction, because they feel that they could handle various teaching tasks well. Many other studies have shown the mediating effects of self-efficacy on employees’ well-being or work engagement, and the subsequent effect on their job satisfaction (Huang et al., 2019). Successful teachers cannot only teach well, but also feel happiness and satisfaction with their work. As a direct variable, self-efficacy has been shown to affect teachers’ job satisfaction, but as an intermediary variable, it has not been discussed in much detail.

### Related Theories of Satisfaction

Job satisfaction has been defined as the affective orientation employees have toward their work (Bishay, 1996; Price, 2001; Skaalvik and Skaalvik, 2010, 2011). Teachers’ satisfaction could be defined as teachers’ affective reactions to their work or to their roles as teachers (Zembylas and Papanastasiou, 2013).

In Porter and Lawler’s (1969) study, internal and external factors that may affect job satisfaction were the focus. They found that internal satisfaction is related to the work itself (including independence and achievement), while external satisfaction does not have a direct relation to the work itself. When teachers have a higher sense of self-efficacy, they have a higher sense of achievement in their teaching work, which then affects their job satisfaction. Wu and Short (1996) found that teachers’ satisfaction could be predicted by self-efficacy and professional growth; that is, the higher the teachers’ self-efficacy, the higher their teachers’ satisfaction will be.

Han et al. (2019) showed that job resources can predict employee well-being through work engagement. Job resources encourage employees’ work engagement and ultimately lead to job satisfaction. Chang et al. (2010) suggested that teaching resources are significantly related to teachers’ teaching efficacy. Moreover, self-efficacy has been found to influence teaching performance (Kao et al., 2011; Telia, 2011). In other words, if suitable resources are provided, teachers’ willingness to teach may increase, and then their self-efficacy and satisfaction may improve. This study proposed a model of online teaching satisfaction, in which SE-OLR and SU-OLR were presented, and TECHN-SE and OT-SE were taken as intermediary variables.

### Research Model and Hypotheses

This study aimed to determine the relationship between the five factors: SE-OLR, SU-OLR, TECHN-SE, OT-SE, and teachers’ satisfaction. Previous studies have shown the relationship between resources and satisfaction. Han et al. (2019) proposed that job resources could encourage teachers’ work engagement and ultimately lead to job satisfaction. In other words, teachers’ specific choices and control of the learning resources may affect teachers’ satisfaction directly. Besides, the suitability of supplied resources and the matching of resources and teaching materials may make teachers feel the usefulness of those resources, thus affecting their online teaching satisfaction. Teachers’ SE-OLR is more about whether teachers can choose resources independently and whether the teaching content can be decided by teachers. Teachers’ SU-OLR relates to whether the use of online educational resources meets their teaching needs. Chang et al. (2010) suggested that teaching resources are significantly related to teachers’ teaching efficacy. SU-OLR and SE-OLR may affect teachers’ self-efficacy. Thus, the following research hypotheses were proposed:

H1: Teachers’ SE-OLR has a positive effect on their OT-SE.
H2: Teachers’ SE-OLR has a positive effect on their TECHN-SE.
H3: Teachers’ SU-OLR has a positive effect on their OT-SE.
H4: Teachers’ SU-OLR has a positive effect on their TECHN-SE.
H7: Teachers’ SE-OLR has a positive effect on teachers’ satisfaction.
H8: Teachers’ SU-OLR has a positive effect on teachers’ satisfaction.

This study considered two types of self-efficacy: TECHN-SE and OT-SE. Teachers’ self-efficacy can be regarded as their estimation and judgment of whether they are competent to perform the task of online education. Moè et al. (2010) proposed that teachers’ self-efficacy could help improve their job satisfaction, and they feel that they could handle various teaching tasks well. Wu and Short (1996) found that job satisfaction among teachers was predicted by self-efficacy. In addition, many other studies have demonstrated the mediating effects of self-efficacy on employees’ well-being or work engagement which then affected their job satisfaction (Huang et al., 2019). Hence, the following research hypotheses were proposed:

H5: Teachers’ OT-SE has a positive effect on teachers’ satisfaction.
H6: Teachers’ TECHN-SE has a positive effect on teachers’ satisfaction.

Chang et al. (2010) showed the correlation between resources and teaching efficacy. Previous studies have shown that self-efficacy could indicate teachers’ job satisfaction (Wu and Short, 1996; Moè et al., 2010). Thus, the two types of self-efficacy (TECHN-SE and OT-SE) may be mediating variables between online learning resources and teachers’ satisfaction. Here were the hypotheses:

H9: Teachers’ SE-OLR is significantly related to teachers’ satisfaction mediated by their OT-SE.
H10: Teachers’ SE-OLR is significantly related to teachers’ satisfaction mediated by their TECHN-SE.
H11: Teachers’ SU-OLR is significantly related to teachers’ satisfaction mediated by their OT-SE.
H12: Teachers’ SU-OLR is significantly related to teachers’ satisfaction mediated by their TECHN-SE.

**FIGURE 1:**
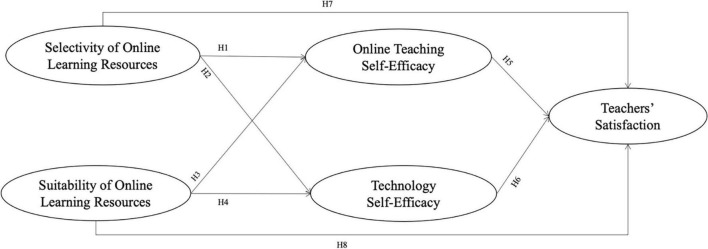
Research model.

The model is illustrated in Figure 1.

## Materials and Methods

### Participants and Data Collection

The participants in this study were teachers from some primary, middle, and high schools in Shanghai, China who carried out online teaching during the COVID-19 epidemic. The questionnaire was sent to a platform for online surveys powered by www.wjx.cn, and was distributed to the participants. The snowball sampling method was used to collect data. A total of 24 teachers from primary, middle, and high school were invited to fill in the questionnaire. These 24 teachers also be invited to help us send questionnaire to their colleagues, and then asked their colleagues to help us send it to other teachers they know. This study distributed a pre-survey questionnaire to teachers, and 50 valid samples were collected. Based on the results of pre-survey analysis and revisions to the questionnaire, a formal questionnaire was used to collect information from a large number of teachers. A total of 1,767 valid questionnaires were finally collected (see Table 1).

**TABLE 1:** Participants’ information.

Variable	Frequency	Percent	Variable	Frequency	Percent	Variable	Frequency	Percent
Gender			Subject			Grade school		
Male	356	20.1%	Chinese	378	21.40%	Primary school	678	38.4%
Female	1411	79.9%	Math	293	16.60%	Middle school	492	27.8%
Total	1767	100%	English	297	16.80%	High school	597	33.8%
			Literature	168	9.50%	Total	1767	100%
			Science	166	9.40%			
			Art	465	26.30%			
			Total	1,767	100.00%			

### Instruments

A questionnaire was designed to test the variables and their relationships, including three parts, the statement part informing the absence of any commercial or any other use of the information, and the voluntary and anonymous participation, the collection part of the participants’ basic information, and the scale part of the variables in the study. The answers to the questionnaire scale were rated on a 5-point Likert scale (1 = very dissatisfied, 2 = relatively dissatisfied, 3 = average, 4 = relatively satisfied, 5 = very satisfied). A professor from a related field was invited to give opinions and suggestions on the accuracy of the language. Based on the suggestions provided, minor changes were made to the wording.

#### Selectivity of Online Learning Resources

We used the revised version of teachers’ selection of online learning resources by Lee and Park (2008), who revised the questionnaire of other scholars (Davis, 1989; Davis et al., 1989; Pavlou, 2003) in order to investigate perceived usefulness and ease of use. Since their study was oriented toward consumers rather than teachers, we further modified the questionnaire to form the items as follows. “Teachers can choose the online learning materials according to the learning content of the textbooks,” “Teachers can adjust the details of the learning content according to the online learning context,” and “Teachers can adjust the difficulty of the learning content.”

#### Suitability of Online Learning Resources

Applying the revised version of teachers’ intention to use online education resources by Chang et al. (2010), the resource suitability for teachers was investigated. Specifically, the items included “I can find certain learning materials with the same teaching progress as my current teaching progress on the internet,” “After simple editing, the learning resources provided online can be successfully applied to my teaching,” and “The materials of online learning resources are the same as the teaching materials I use in my instruction without any revision.”

#### Technology Self-Efficacy

In this study, the questionnaire was modified in combination with the scale of Huang et al. (2019), and the definition of TECHN-SE (Compeau and Higgins, 1995). The TECHN-SE scale contained items such as, “I think the school internet equipment is sufficient and can meet my use requirements at any time,” “I think the school gives me sufficient permission to use the online learning resources required for teaching,” and “When I want to use the online learning resources, I will not delay my use due to equipment, the internet or other reasons.”

#### Online Teaching Self-Efficacy

In this study, the questionnaire was modified in combination with the scale of Huang et al. (2019), and the definition of OT-SE (Li, 2019). The TECHN-SE scale included items such as, “I can better design teaching with the help of communication tools,” “I can carry out and exit various online teaching applications smoothly,” “I can use the computer to search for necessary information and download software,” and “I can use the computer for word, graphics and images, and video processing.”

#### Teachers’ Satisfaction

The questionnaire developed by Wu and Short (1996) was modified in this study to assess teachers’ satisfaction. The following items were the examples: “I am willing to use online teaching resources frequently,” “I am willing to try more online teaching methods in teaching,” and “After COVID-19, I will continue to apply online teaching in my instruction if I can.”

### Data Analysis

To verify the hypotheses, this study used IBM SPSS (version 26.0) and IBM AMOS (version 26.0) to conduct exploratory factor analysis (EFA) and confirmation factor analysis (CFA).

First, this study conducted EFA to verify the applicability of the scale. The independent sample *t* test was used to ensure that each item had good discrimination. 1 item with poor discrimination was deleted. The results showed that there was a significant difference between the high and low groups. EFA was used to verify the structure validity of the scale. The value of KMO equal to 0.962 > 0.7, and the Barlett sphere test (*p* < 0.001) met the recommended values (Watkins, 2021). Principal component analysis and maximum variance analysis were then used to extract factors. 2 items had a factor loading coefficient below 0.5 were deleted. A total of four factors with characteristic values greater than 1 were generated. The cumulative variance explanation rate of the five factors extracted by factor analysis was 78.52%, indicating that the extracted five factors met the recommended value (>70%) (Watkins, 2021). The consistency coefficient value of the five factors was 0.921, indicating that each scale had a reliable internal reliability. Thus, 16 items were finally retained for a formal survey of online education satisfaction.

To confirm the item suitability of the measuring questionnaire, first-order CFA was used in this study. Moreover, model-fit indexes of the measurement items were used to examine the measurement model. Structural equation modeling was used to assess the hypothetical structural model.

## Results

### Item Analysis

The reason for performing item analysis is to ascertain whether questionnaire items are effective and appropriate. Each item (A1-F3, A1-A3 are items of SE-OLR; B1-B3 are items of SU-OLR; C1-C3 are items of TECHN-SE; D1-D4 are items of OT-SE; F1-F3 are items of teachers’ satisfaction.) in the scale was analyzed using the 27/73 quantile method. The principle is to first sum up the analysis items, then divide them into high scoring and low scoring groups, followed by performing a *t* test to compare the two groups. If differences are found, it indicates an appropriate proportional item design; if not, items should be deleted. Table 2 shows that 16 items of A1- F3 were analyzed, and after summing these 16 items, they were divided into high and low groups, and a *t* test was performed to identify any differences.

**TABLE 2:** Item analysis.

	Group (Mean ± SD)		*t*
	Low group (*n* = 488)	High group (*n* = 494)	
A1	3.80 ± 0.80	4.94 ± 0.25	−30.056***
A2	3.61 ± 0.86	4.90 ± 0.33	−31.142***
A3	3.77 ± 0.78	4.95 ± 0.22	−31.999***
B1	3.32 ± 0.84	4.90 ± 0.30	−39.221***
B2	3.14 ± 0.84	4.91 ± 0.30	−43.479***
B3	3.32 ± 0.96	4.88 ± 0.33	−33.8***
C1	3.18 ± 0.93	4.87 ± 0.36	−37.5***
C2	3.43 ± 0.97	4.91 ± 0.29	−32.421***
C3	2.86 ± 1.12	4.74 ± 0.65	−32.126***
D1	3.27 ± 0.96	4.80 ± 0.40	−32.522***
D2	3.61 ± 0.84	4.93 ± 0.26	−32.988***
D3	3.35 ± 0.94	4.82 ± 0.45	−31.134***
D4	3.52 ± 0.77	4.94 ± 0.25	−38.79***
F1	3.11 ± 0.92	4.91 ± 0.29	−41.656***
F2	3.32 ± 0.88	4.95 ± 0.24	−39.281***
F3	2.95 ± 0.84	4.67 ± 0.65	−36.124***

*** indicates significance when p < 0.001.

The high and low groups showed significant differences for A1-F3 (*p* < 0.001), which indicated that a total of 16 items had good discrimination and should be retained (see Table 2). Consequently, a total of 16 items were well differentiated, and the analysis items did not need to be removed.

### Reliability and Validity Analysis

#### Reliability Analysis

Testing reliability can also be called reliability analysis, which determines whether the answers to the questionnaire are consistent. An alpha coefficient greater than 0.6 indicates good consistency with Cronbach’s alpha. SPSS25.0 was used to test the reliability of the questionnaire data. There was a high level of reliability for each component of the dimension (see Table 3), which meant that reliability for each component met the standards of the study; that is, the construct validity was good.

**TABLE 3:** Reliability of the model.

Dimension	*N*	Cronbach’s Alpha
SE-OLR	3	0.931
SU-OLR	3	0.868
TECHN-SE	3	0.790
OT-SE	4	0.890
Teachers’ satisfaction	3	0.866

#### Validity Analysis

The KMO measurement value was greater than 0.8. The approximate chi square value of the Bartley spherical test was 3,307.585, the degree of freedom was 153, and the *p* value was less than 0.001. It can thus be seen that the online education satisfaction scale was suitable for factor analysis.

### Model Fit Analysis

In the process of model training, overfitting is a very common phenomenon due to the large sample size. The overfitting means that it performs well on the training set, but performs poorly on the test set. In order to reduce overfitting and improve the generalization ability of the model, there are many measures to alleviate the problem of overfitting in practice. One of the common methods is cross validation (James et al., 2013). Prior studies have applied this method (Islam et al., 2015; Alt and Itzkovich, 2017; Rossin and Bergee, 2020). Therefore, we divided the data into two groups. The first group of data is 895, and the second group of data is 872. We used group 1 to establish the model, and the results are shown in Table 4. We then used group 2 for cross validation, and the results are shown in Table 5.

**TABLE 4:** Measurement model results (Group 1, *n* = 895).

	CR	Alpha	AVE	SE-OLR	SU-OLR	TECHN-SE	OT-SE	Teachers’ Satisfaction
SE-OLR	0.819	0.931	0.931	**0.905**				
SU-OLR	0.693	0.870	0.871	0.575	**0.833**			
TECHN-SE	0.556	0.776	0.789	0.559	0.686	**0.746**		
OT-SE	0.657	0.884	0.884	0.568	0.655	0.651	**0.811**	
Teachers’ satisfaction	0.688	0.863	0.868	0.629	0.661	0.656	0.697	**0.829**

*A bold number on the diagonal represents the square root of the variance shared between the constructs and their measures. A correlation between two constructs is an off-diagonal element. A diagonal element must be larger than a non-diagonal element if discriminate validity is to be achieved.*

**TABLE 5:** Measurement model results (Group 2, *n* = 872).

	CR	Alpha	AVE	SE-OLR	SU-OLR	TECHN-SE	OT-SE	Teachers’ Satisfaction
SE-OLR	0.932	0.931	0.822	**0.906**				
SU-OLR	0.866	0.865	0.684	0.584	**0.827**			
TECHN-SE	0.813	0.803	0.592	0.539	0.704	**0.77**		
OT-SE	0.896	0.896	0.683	0.561	0.699	0.669	**0.826**	
Teachers’ Satisfaction	0.871	0.869	0.692	0.656	0.705	0.702	0.719	**0.832**

*A bold number on the diagonal represents the square root of the variance shared between the constructs and their measures. A correlation between two constructs is an off-diagonal element. A diagonal element must be larger than a non-diagonal element if discriminate validity is to be achieved.*

The validity test results showed that the chi square to degree of freedom ratio was 4.475 (χ^2^/*df* < 5), the mean square sum of asymptotic residuals (RMSEA) was 0.064 (RMSEA < 0.08), GFI was 0.939, NFI was 0.959, CFI was 0.968, and IFI was 0.968. The above inspection indexes generally met the standard of ideal fitting index values. At the same time, in the model parameter test, there was no abnormal value in the standardization error of the model (S.E. > 0), and the significance test of the parameters met the standard range (C.R. > 2, *p* < 0.001), indicating that the validity of this model was good.

This questionnaire had a Cronbach’s alpha coefficient that ranged from 0.776 to 0.931, which meant that it was highly reliable. The combined reliability CR value for the model also met the requirements, as it represents the internal consistency of the variables and, accordingly, indicates that the sample data which were collected met the requirements for internal consistency. A test for validity revealed that the average variance extraction ratio (AVE) across all measures was above 0.5, supporting the idea that observed variables could explain the measurement dimension well. AVE ratios of the measurement’s dimensions were greater than the correlation coefficients of these measurements and the others, showing that the measurement model excelled in discriminating between them (see Table 4).

The validity test results showed that the chi square to degree of freedom ratio was 4.353 (X^2^/*df* < 5), the mean square sum of asymptotic residuals (RMSEA) was 0.061 (RMSEA < 0.08), GFI was 0.945, NFI was 0.965, CFI was 0.973, and IFI was 0.973. The above inspection indexes generally met the standard of ideal fitting index value. At the same time, in the model parameter test, there was no abnormal value in the standardization error of the model (S.E. > 0), and the significance test of the parameters met the standard range (C.R. > 2, *p* < 0.001), indicating that the validity of this model was high.

This questionnaire had a Cronbach’s alpha coefficient that ranged from 0.803 to 0.931, which means that it was highly reliable. The combined reliability CR value for the model also met the requirements, as it represents the internal consistency of the variables and, accordingly, indicates that the sample data collected met the requirements for internal consistency. A test for validity revealed that the average variance extraction ratio (AVE) across all measures is above 0.5, supporting the idea that observed variables could well explain the measurement dimension. AVE ratios of the measurement’s dimensions were greater than the correlation coefficients of these measurements and the others, showing that the measurement model excelled in discriminating between them (see Table 5).

Additionally, there is no multicollinearity between independent variables. The tolerance of explanatory variables (1/VIF) in the model is greater than 0.10 and the variance expansion factor (VIF) is less than 3, indicating that there is no multicollinearity between variables and it will not affect the correct estimation of the model (Thompson et al., 2017) (see Table 6).

**TABLE 6:** Variance inflation factor results.

Variables	VIF	1/VIF
SE-OLR	1.684	0.594
SU-OLR	2.448	0.409
TECHN-SE	2.280	0.439
OT-SE	2.230	0.448

### Path Analysis

**FIGURE 2:**
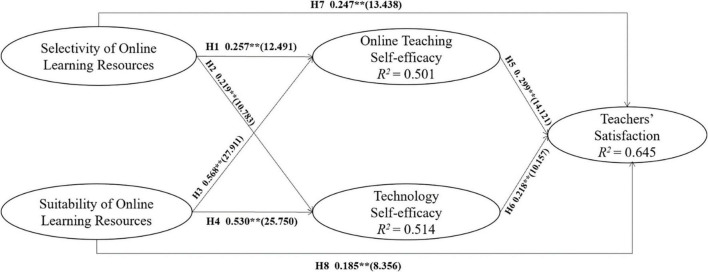
Model results. ** indicates significance at *p* < 0.01.

Four models were established to test the mediating effect of the variables. Model 1 included SE-OLR, SU-OLR, and teachers’ satisfaction. Model 2 added OT-SE to Model 1 to test its mediating role. Model 3 added TECHN-SE to Model 1 to test the mediating role of TECHN-SE. On the basis of Model 1, Model 4 added OT-SE and TECHN-SE to test the mediating effect of OT-SE and TECHN-SE on SE-OLR, SU-OLR, and TS (see Figure 2). *R*^2^ value represents a quantity called the coefficient of determination, which is defined as the proportion of variation in the response variable that is accounted for by a regression model and any explanatory variables with which it is associated. *R*^2^ values higher than 0.6 indicate that there is a high correlation between variables, *R*^2^ values between 0.3 and 0.6 indicate that there is a medium correlation between variables, and *R*^2^ values less than 0.3 are considered low (Barrett, 2000; Sanchez, 2013). As shown in Figure 2, SE-OLR and SU-OLR had a medium impact on OT-SE and TECHN-SE, and OT-SE and TECHN-SE had a high impact on teachers’ satisfaction.

In Model 4, SE-OLR and SU-OLR significantly affected OT-SE and TECHN-SE, and so H1 (β = 0.257, *t* = 12.491**), H2 (β = 0.219, *t* = 10.783**), H3 (β = 0.568, *t* = 27.911**), and H4 (β = 0.530, *t* = 25.750**) were all supported (see Table 7).

**TABLE 7:** Hypothesis test analysis results.

Assumption	β	*t*	Supported or not
H1: SE-OLR of teachers has a positive effect on their OT-SE.	0.257	12.491**	Yes
H2: SE-OLR of teachers has a positive effect on their TECHN-SE.	0.219	10.783**	Yes
H3: SU-OLR of teachers has a positive effect on their OT-SE.	0.568	27.911**	Yes
H4 SU-OLR of teachers has a positive effect on their TECHN-SE.	0.530	25.750**	Yes
H5: OT-SE of teachers has a positive effect on teachers’ satisfaction.	0.299	14.121**	Yes
H6 TECHN-SE of teachers has a positive effect on their teachers’ satisfaction.	0.218	10.157**	Yes
H7: SE-OLR of teachers has a positive effect on teachers’ satisfaction.	0.247	13.438**	Yes
H8: SU-OLR of teachers has a positive effect on teachers’ satisfaction.	0.185	8.356**	Yes
H9: SE-OLR is significantly related to satisfaction mediated by OT-SE.	–	–	Yes
H10: SE-OLR is significantly related to satisfaction mediated by TECHN-SE.	–	–	Yes
H11: SU-OLR is significantly related to satisfaction mediated by OT-SE.	–	–	Yes
H12: SU-OLR is significantly related to satisfaction mediated by TECHN-SE.	–	–	Yes

** indicates significance at p < 0.01.

In Model 1, SE-OLR and SU-OLR significantly affected teachers’ satisfaction. H5 (β = 0.299, *t* = 14.121**), H6 (β = 0.218, *t* = 10.157**), H7 (β = 0.247, *t* = 13.438**), and H8 (β = 0.185, *t* = 8.356**) were therefore all supported (see Table 7).

### Indirect Effect

As shown in Table 8, since OT-SE was used as the mediation variable of SE-OLR and teachers’ satisfaction, and c’ and a × b were both significant, there was a partial mediation effect. TECHN-SE was used as the mediation variable of SE-OLR and teachers’ satisfaction, and c’ and a × b were both significant; thus, there was a partial mediation effect. Therefore, H9 and H10 were supported.

**TABLE 8:** Mediation test results.

Term	c Total Effect	a	b	a × b Mediating Effect	a × b (95% BootCI)	c’ Direct Effect	Test conclusion
SE-OLR ≥ TECHN-SE ≥ Teachers’ Satisfaction	0.436**	0.260**	0.215**	0.056	0.031 ∼ 0.065	0.290**	Partial mediation
SE-OLR ≥ OT-SE ≥ Teachers’ Satisfaction	0.436**	0.264**	0.342**	0.09	0.056 ∼ 0.100	0.290**	Partial mediation
SU-OLR ≥ TECHN-SE ≥ Teachers’ Satisfaction	0.482**	0.591**	0.215**	0.127	0.091 ∼ 0.160	0.191**	Partial mediation
SU-OLR ≥ OT-SE ≥ Teachers’ Satisfaction	0.482**	0.478**	0.342**	0.163	0.127 ∼ 0.194	0.191**	Partial mediation

** indicates significance at p < 0.01.

OT-SE was used as the mediating variable of sustainability of SU-OLR and teachers’ satisfaction, and c’ and a × b were both significant; thus, there was a partial mediation effect. TECHN-SE was used as the mediation variable of the feasibility of SU-OLR and teachers’ satisfaction, and c’ and a × b were both significant, so there was a partial mediation effect. Therefore, H11 and H12 were supported.

## Discussion

The results showed that SE-OLR had a positive effect on OT-SE and TECHN-SE, indicating that H1 and H2 were supported. It showed that with the improvement of SE-OLR, teachers’ OT-SE and TECHN-SE will also be enhanced. The results showed that SU-OLR had a positive effect on OT-SE and TECHN-SE, indicating that H3 and H4 were supported. It showed that with the improvement of SU-OLR, teachers’ OT-SE and TECHN-SE will also be enhanced. According to the study of Chang et al. (2010), learning resources were significantly related to teachers’ teaching efficacy, which is consistent with the findings of this study. Timothy (2009) correlated TECHN-SE with online learning resources, and concluded that the improvement of TECHN-SE could make online learning resources more fully utilized. However, his study did not explore the effect of online learning resources on TECHN-SE. This study mentioned it and found that online learning resources can have a positive impact on OT-SE and TECHN-SE.

The results showed that SE-OLR and SU-OLR had a positive effect on teachers’ satisfaction, indicating that H7 and H8 were supported. It showed that with the improvement of SE-OLR and SU-OLR, teachers’ satisfaction will also increase. Mulhem and Wang (2020) showed that course content quality had a positive effect on satisfaction. In addition, Han et al. (2019) examined the relationship between teachers’ job resources and their job satisfaction. They found that job resources could improve teachers’ happiness in the process of work, thus encouraging them to participate in work and ultimately leading to higher job satisfaction. These conclusions are also reflected in this study, and as the main attributes of online learning resources were divided in this study, it makes the ways of promoting the role of online learning resources in teachers’ satisfaction more clearly.

The results showed that OT-SE and TECHN-SE had a positive effect on teachers’ satisfaction, indicating that H5 and H6 were supported. It showed that with the improvement of teachers’ OT-SE and TECHN-SE, teachers’ satisfaction will also increase. Wu and Short (1996) mentioned that the improvement of teachers’ self-efficacy contributed to the improvement of TS. Moè et al. (2010) believed that when teachers have a high sense of self-efficacy, they will be more confident that they can handle a variety of teaching tasks, and teachers’ satisfaction will increase accordingly. Moreover, many other studies have demonstrated the effects of self-efficacy on employees’ well-being or work engagement and then this will affect teachers’ satisfaction (Huang et al., 2019), which is consistent with the findings of this study.

The results showed that SE-OLR and SU-OLR, mediated by OT-SE and TECHN-SE, had a positive impact on teachers’ satisfaction, indicating that H9, H10, H11, and H12 were supported. It showed that with the improvement of SE-OLR and SU-OLR, teachers’ OT-SE and TECHN-SE will increase, which will lead to the improvement of teachers’ satisfaction. In previous studies, Chang et al. (2010) showed the correlation between resources and teachers’ self-efficacy, and studies by Moè et al. (2010) and Wu and Short (1996) showed that self-efficacy could reflect teachers’ job satisfaction. Therefore, self-efficacy can act as an intermediary to affect satisfaction in a process, which was also confirmed in this study.

## Conclusion

Online learning resources play an important role in teaching and learning in the process of online learning during the COVID-19 lockdown. This study considered the mediating role of teachers’ self-efficacy, and explored the relationship between online learning resources and teachers’ satisfaction. The results indicated that SE-OLR and SU-OLR, and TECHN-SE and OT-SE are positively related to teachers’ satisfaction, while TECHN-SE and OT-SE play mediating roles between SE-OLR and SU-OLR and teachers’ satisfaction.

### Implications

For online teaching, this study is of importance both theoretically and practically. At the theoretical level, teachers’ satisfaction directly influences online learning effectiveness. Exploring the factors which affect teachers’ satisfaction will be of great help to improve online learning effectiveness. The study investigated the relationship between online learning resources and teachers’ satisfaction. The results showed that the selectivity and suitability of online learning resources can affect teachers’ satisfaction by affecting their self-efficacy, with teachers’ self-efficacy playing the mediating role. The findings reveal the relationship of online learning resources and teachers’ satisfaction for OLR developers and teachers to figure out the key points of improving online learning resources for online learners.

At a practical level, with the sudden transition to online teaching, there is not sufficient suitable and useful online learning resources to support teachers to conduct their teaching activities. Teachers need to select some online learning resources and integrate them as suitable learning resources provided to online learners. The findings will lead OLR designers, developers, and teachers to pay attention to the promotion of online learning resources and teachers’ self-efficacy to fulfill the requirements of online teachers in order to eventually improve online learners’ learning effectiveness.

### Limitations and Future Study

Despite the useful findings, this study also has some limitations. First, the sample is from a specific area and the results are limited to the target area and individuals and groups. Secondly, the teachers had not prepared well or adapted to online education. In the future, with the development of online education, the impact of regional differences on online learning resources and teachers’ satisfaction needs to be further verified to ensure the applicability of the conclusion proposed by the study.

## Data Availability Statement

The original contributions presented in the study are included in the article/supplementary material, further inquiries can be directed to the corresponding author/s.

## Author Contributions

All authors contributed equally to the conception of the idea, implementing and analyzing the experimental results, writing the manuscript, and reading and approving the final manuscript.

## Conflict of Interest

The authors declare that the research was conducted in the absence of any commercial or financial relationships that could be construed as a potential conflict of interest.

## Publisher’s Note

All claims expressed in this article are solely those of the authors and do not necessarily represent those of their affiliated organizations, or those of the publisher, the editors and the reviewers. Any product that may be evaluated in this article, or claim that may be made by its manufacturer, is not guaranteed or endorsed by the publisher.
